# Better evaluation of PTSD by MRI

**Published:** 2012-11

**Authors:** Hamid Reza Saeidiborojeni, Elham Shobeiri, Zahra Aslani, Sepehr Saeidiborojeni

**Affiliations:** ^*a*^Department of Neurosurgery , Kermanshah University of Medical Sciences, Kermanshah, Iran.; ^*b*^Department of Radiology , Kermanshah University of Medical Sciences, Kermanshah, Iran.; ^*c*^Tehran University of Medical Sciences, Tehran, Iran.

## Abstract

**Background:**

Traumatic brain injury (TBI) is a common problem worldwide that most of traumatic patients suffer from mental and cognitive disorders. The most common trait of this disorder, defined by computed tomography (CT) scan is small hemorrhagic lesions in cerebral white matter of the patient. These hemorrhagic lesions seem to be an effective factor for occurrence of neuropsychiatric symptoms in Post Traumatic Stress Disorder patients. So far, several diagnostic modalities such as T2GE and T2SE have been employed to detect these lesions. The present study aims to compare the diagnostic efficacy of these two sequences for detection of brain hemorrhagic lesions in patients with a history of head trauma that have neuropsychiatric symptoms. Abnormal findings in the images obtained by T2SE and T2GE sequences of Magnetic Resonance Imaging (MRI) in chronic traumatic brain lesions.

**Methods:**

Thirty patients with a history of head trauma, referred to neurosurgery department because of neuropsychiatric symptoms (PTSD) were prospectively assessed using MRI. Then, the correlation between the findings of T2GE and T2SE sequences and patient’s clinical symptoms were analyzed by Wilcoxon Signed Ranks Test (all the patients had these symptoms at least three months after head trauma).

**Results:**

The T2GE sequence showed a significant higher diagnostic efficacy (p less than 0/01) indicating 80% of patients had hemorrhagic lesions, compared to theT2SE sequence detecting in just 23% of patients. Therefore, the T2GE is significantly better than T2SE in detecting the cause of clinical symptoms in traumatic patients (p less than 0/01).

**Conclusions:**

The T2GE sequence showed a higher diagnostic efficacy, compared to the T2SE, for evaluating the neuropsychiatric symptoms in chronic traumatic patients with a previous history of head trauma. So this sequence could play a major role in diagnosis and treatment of PTSD patients especially in their legal problems. Based on the findings of the present study, it is recommended that T2GE sequence of MRI can be a reliable method for evaluation of chronic traumatic patients.

**Keywords:**

Traumatic brain injury, MRI, T2GE, T2SE


					Volume 4, Suppl. 1 Nov 2012
Publisher: Kermanshah University of Medical Sciences
URL: http://www.jivresearch.org
Copyright:
Congress of Iranian Neurosurgeons, 14-16 November 2012, Kermanshah, Iran
Paper No. 12
Better evaluation of PTSD by MRI
Hamid Reza Saeidiborojeni a
, Elham Shobeiri b,*
, Zahra Aslanib
, Sepehr Saeidiborojeni c
a
Department of Neurosurgery, Kermanshah University of Medical Sciences, Kermanshah, Iran.
b
Department of Radiology, Kermanshah University of Medical Sciences, Kermanshah, Iran.
c
Tehran University of Medical Sciences, Tehran, Iran.
Abstract:
Background: Traumatic brain injury (TBI) is a common problem worldwide that most of traumatic patients suffer from mental and
cognitive disorders. The most common trait of this disorder, defined by computed tomography (CT) scan is small hemorrhagic
lesions in cerebral white matter of the patient. These hemorrhagic lesions seem to be an effective factor for occurrence of
neuropsychiatric symptoms in Post Traumatic Stress Disorder patients. So far, several diagnostic modalities such as T2GE and T2SE
have been employed to detect these lesions. The present study aims to compare the diagnostic efficacy of these two sequences for
detection of brain hemorrhagic lesions in patients with a history of head trauma that have neuropsychiatric symptoms. Abnormal
findings in the images obtained by T2SE and T2GE sequences of Magnetic Resonance Imaging (MRI) in chronic traumatic brain
lesions.
Methods: Thirty patients with a history of head trauma, referred to neurosurgery department because of neuropsychiatric
symptoms (PTSD) were prospectively assessed using MRI. Then, the correlation between the findings of T2GE and T2SE sequences
and patient's clinical symptoms were analyzed by Wilcoxon Signed Ranks Test (all the patients had these symptoms at least three
months after head trauma).
Results: The T2GE sequence showed a significant higher diagnostic efficacy (p less than 0/01) indicating 80% of patients had
hemorrhagic lesions, compared to theT2SE sequence detecting in just 23% of patients.
Therefore, the T2GE is significantly better than T2SE in detecting the cause of clinical symptoms in traumatic patients (p less than
0/01).
Conclusions: The T2GE sequence showed a higher diagnostic efficacy, compared to the T2SE, for evaluating the neuropsychiatric
symptoms in chronic traumatic patients with a previous history of head trauma. So this sequence could play a major role in
diagnosis and treatment of PTSD patients especially in their legal problems. Based on the findings of the present study, it is
recommended that T2GE sequence of MRI can be a reliable method for evaluation of chronic traumatic patients.
Keywords:
Traumatic brain injury, MRI, T2GE, T2SE
* Corresponding Author at:
Elham Shobeiri: Department of Radiology, Kermanshah University of Medical Sciences, Kermanshah, Iran, Tel: 09181310050,
Email: elhamshobeiri@gmail.com, (Shobeiri E.).

				

